# Comparison of a gelatin thrombin versus a modified absorbable polymer as a unique treatment for severe hepatic hemorrhage in swine

**DOI:** 10.1038/s41598-023-41983-9

**Published:** 2023-11-27

**Authors:** Francisco José Sánchez del Valle, Luis De Nicolás, Guillermo Fernández, Pedro Fernández, Esther Gómez, Inmaculada Aranaz Corral

**Affiliations:** 1 Central Hospital of Defense, General and Digestive Unit, Spanish Ministry of Defense, Glorieta del Ejército, 1, 28047 Madrid, Spain; 2 Central Hospital of Defense, Unit of Surgical Research, Spanish Ministry of Defense, Madrid, Spain; 3 University of Alcalá de Henares, Madrid, Spain; 4 Complutense University, Madrid, Spain

**Keywords:** Liver, Preclinical research

## Abstract

There are many surgical techniques (packing, Pringle maneuver, etc.) and hemostatic agents to manage hepatic bleeding in trauma surgery. This study compares the effectiveness of two different types of hemostatic agents, one is an active flowable hemostat and the other is a passive hemostat made of modified absorbable polymers [MAP]. Both surgical technique and hemostatic agents can be used together as a means of controlling bleeding. We have hypothesized that a single hemostatic agent might be as effective as a unique hemostatic surgical technique. Twenty swine were prospectively randomized to receive either active Flowable (Floseal) or passive MAP powder (PerClot) hemostatic agents. We used a novel severe liver injury model that caused exsanguinating hemorrhage. The main outcome measure was total blood loss volume. The total volume of blood loss, from hepatic injury to minute 120, was significantly lower in the Flowable group (407.5 cm^3^; IqR: 195.0–805.0 cm^3^) compared to MAP group (1107.5 cm^3^; IqR: 822.5 to 1544.5 cm^3^) (Hodges–Lehmann median difference: − 645.0 cm^3^; 95% CI: − 1144.0 to − 280.0 cm^3^; p = 0.0087). The rate of blood loss was significantly lower in the flowable group compared with the MAP group as measured from time of injury to minutes 3, 9, 12, and 120 (except for 6 min). The mean arterial pressure gradually recovered in the flowable group by 24 h, whereas in the MAP group, the mean arterial pressure was consistently stayed below baseline values. Kaplan–Meier survival analysis indicated similar rates of death between study groups (Logrank test p = 0.3395). Both the flowable and the MAP hemostatic agents were able to effectively control surgical bleeding in a novel severe liver injury model, however, the flowable gelatin–thrombin agent provided quicker and better bleed control.

## Introduction

Bleeding is a prevalent complication of liver surgery that negatively affects clinical outcomes. Surgical bleeding of the liver can lead to a significant increase in morbidity and mortality rates, longer surgical procedures, longer hospital stays, and increased costs^1–3^. Therefore, reducing surgical bleeding may positively impact clinical and economic aspects^1,4^. Different strategies have been used to reduce surgical bleeding, specifically, hemostatic products have been used and classified in different ways based on their mechanism of action. One of the most widely used classifications for hemostatic products is based on whether the product provides a physical structure around which platelets can aggregate to form a clot (passive) or whether the product delivers its mechanism of action on the clotting cascade in a biologically active manner (active)^5^. Among these, topical hemostatic agents have been used to improve surgical hemostasis^1,6^.

Currently, various hemostatic agents are available as adjunctive measures to control surgical bleeding. Some active agents, including fibrinogen and thrombin, actively participate at the end of the coagulation cascade to form fibrin clots^7–9^. These agents can be effectively used in patients with spontaneous or drug-induced coagulation disorders and are effective for a wide range of bleeding rates (including pulsatile arterial bleeding)^5,9^. Others, such as porcine gelatin^10^, oxidized cellulose^11^, and plant-derived polysaccharide spheres^12^ are known as passive hemostatic agents. They activate and aggregate platelets and form a matrix at the site of bleeding, allowing clotting to occur^9^. Their efficacy depends on the patient’s own fibrin production to achieve hemostasis, so they are only appropriate for patients who have an intact coagulation system^9–12^.

In previous studies, we investigated the ease of use and strength of a novel modified absorbable polymer (MAP) powder (PerClot) compared with a surgical technique^13^. The purpose of the current study was to compare this novel MAP powder hemostatic agent with a well-known gelatin–thrombin flowable product, as a single hemostatic treatment, without any other surgical technique, in the same severe experimental liver hemorrhage model.

## Materials and methods

### Study design

This prospective, randomized, and experimental study was conducted on 20 female swine (Large White) in the Surgical Research Unit of the Hospital Central de la Defensa “Gómez Ulla” (Madrid, Spain).

The study protocol (Register number: ES280790000187) was approved by the Ethics Committee, Hospital Central de la Defensa Education Committee, and Council of the Environment of the Community of Madrid. The study was conducted in accordance with the Spanish and European legislation regarding animal experimentation, and all methods were reported in accordance with the ARRIVE guidelines. At the end of the study (24 h after the procedure), the animals were euthanized with an anesthetic overdose in accordance with the current legislation.

### Study animals

Twenty healthy female swine (Sus corfa, Large White), with an average weight of 35.5 kg (33.5–40.0 kg), underwent a quarantine period, and a veterinary examination to rule out the presence of any underlying disease prior to study start.

### Study groups

Animals were randomly assigned to the Flowable group receiving treatment with a gelatin–thrombin flowable hemostatic agent (Floseal, Baxter Healthcare Corporation Hayward, CA, USA) utilizing two 5 mL syringes, for a total of 10 mL or the MAP group receiving a MAP powder hemostatic agent (PerClot, Polysaccharide Hemostatic System, Baxter Healthcare Corporation Deerfield, IL, USA) receiving two packages of 5 g each, with a total of 10 g.

### Procedures

#### Anesthetic procedure and monitoring

Anesthesia (atropine sulfate, azaperone, midazolam, ketamine 2%, sevoflurane, fentanyl, and phenylephrine) and fluids were administered via a venous line. A tube ranging from 6.5 to 7.5 mm connected to a ventilator (rate 12–15 breaths/min), was used to intubate the animals.

The animals were monitored using electrocardiography, pulse oximetry, vaginal temperature probe, and capnography. In addition, a femoral arterial probe was used for the invasive monitoring of blood pressure and heart rate.

Balanced saline solution was administered prior to the surgical procedure in order to assure both groups were equally hydrated.

#### Surgical procedure

After performing an extended right subcostal laparotomy, the middle (segment IV) and left (segments II and III) suprahepatic veins were located using echo-Doppler (Logic V2; General Electric, Chicago, IL, USA), where two incisions, 2 cm long and 5 cm deep, were made on the liver parenchyma using a No. 20 scalpel blade. Post-mortem, the investigators verified that both veins had been completely sectioned.

After injury, hemostatic agents were applied according to the manufacturer’s instructions^14,15^. Two 5 mL syringes, with a total of 10 mL of gelatin–thrombin flowable hemostatic agent, were applied. For MAP, two packages of 5 g each for a total of 10 g were applied directly, applying pressure to the injury site for 3 min. No further product or compression was applied, and hemostasis was evaluated at 6, 9, 12, 60, and 120 min.

In this method, hemostatic agents were prepared prior to hepatic injury, to ensure the time required to apply the hemostatic compound was consistent for both groups.

Ringer Lactate Solution (RLS) was administered to restore hydration and fluid balance. To equalize the volume of administered RLS to the measured blood lost by the liver injury, RLS fluid was given at a rate of 1000 ml/h, starting after hepatic injury to minute 12. This was followed by a 500 ml/h infusion from minute 13 to minute 60. For the time period between minute 61 and minute 120, the rate of RLS administration was calculated in order to complete the reposition volume. By minute 120, the animals received an equal volume of RLS in accordance with how much blood was lost.

The blood was removed using a surgical aspirator (Flexivac^®^) and gauze packing pads. The volume of blood loss was calculated using the following formula: “v = [(b_1_ − a_1_) + (b_2_ − a_2_)]/1.04”, where “b_1_” was the weight of the tank of the surgical aspirator loaded with blood, “a_1_” was the dry weight of the tank (without blood), “b_2_” was the weight of the surgical pads soaked in blood,“a_2_” was the dry weight of the surgical pads (without blood) and 1.04 was the numerical constant representing the blood density of the swine model^16^.

### Outcomes

#### Primary outcome

The primary endpoint was measured as the total volume of blood lost in the time period after hepatic injury to minute 120.

#### Secondary outcomes

Secondary outcome measures included: the proportion of animals in which hemostasis was achieved at minute 3, 6, 9, 12, 60, and 120, heart rate (beats per minute, bpm), mean arterial pressure, and survival rate at 120 min.

The amount of time required to apply the hemostatic product was recorded and used as an indicator to measure the difficulty in applying the hemostatic agent. It was assumed that a longer application time correlated with a greater degree of difficulty.

### Statistical analysis

A standard statistical analysis was performed using MedCalc^®^ Statistical Software version 20.218 (MedCalc Software Ltd, Ostend, Belgium; https://www.medcalc.org; 2023).

Descriptive statistics such as number (percentage) and median (interquartile range, IqR) were used as appropriate.

Intragroup comparisons of blood loss and hemodynamic parameters were performed using the Friedman’s two-way analysis test.

The Mann–Whitney *U* test was used to compare the different parameters between the flowable and MAP groups.

Survival rates were plotted for the study groups using Kaplan–Meier analysis and compared using a Log-rank test.

Linear regression analysis was used to assess the relationship between the time of application (independent variable) and the volume of blood lost at 120 min (dependent variable).

Categorical variables were compared using the chi-square test and Fisher’s exact test, as needed.

A p-value of less than 0.05 was considered significant. Animals that died during the study were entered as 0 to denote a censored observation.

### Ethical approval

The study protocol (Register number: ES280790000187) was approved by the Ethics Committee, Hospital Central de la Defensa Education Committee, and Council of the Environment of the Community of Madrid. The study was conducted in accordance with the Spanish and European legislation regarding animal experimentation, and all methods were reported in accordance with the ARRIVE guidelines.

## Results

### Preoperative values

Twenty-four female swine were enrolled in the study, but four of the flowable group animals did not pass the quarantine. Eight (40.0%) pigs in the flowable group and twelve (60.0%) pigs in the MAP group were included in the study. In the overall study sample, the median (IqR) body weight was 35.5 kg (range: 33.5–40.0 kg), with no significant differences between groups (Hodges–Lehmann median difference: 3.3 kg; 95% confidence interval: − 2.5 to 9.5 kg; p < 0.2022). The baseline clinical characteristics of the animals are summarized in Table 1. Briefly, the baseline variables were comparable between groups, with the exception of the volume of balanced saline solution administered, which was significantly greater in the MAP group (Hodges–Lehmann median difference: 167.5 cm^3^; 95% CI: 89.0–306.7 cm^3^; p < 0.0009) (Table 1).

**Table 1:** A comparison of the main baseline clinical between the gelatin–thrombin flowable and the modified absorbable polymer (MAP) groups. Statistical significance was calculated by Mann–Whitney *U* test.

Variable	Flowable (n = 8)	MAP (n = 12)	p
Weight, kg			
Median (IqR)	34.3 (28.0–38.3)	36.5 (34.0–41.5)	0.2022
Heart rate, bpm			
Median (IqR)	82.0 (69.0–103.5)	78.0 (75.0–93.0)	0.9692
MBP*, mmHg			
Median (IqR)	70.5 (63.5–78.0)	70.5 (67.5–77.5)	0.9078
Hemoglobin, g/dL			
Median (IqR)	9.3 (8.4–10.4)	8.5 (8.2–9.0)	0.1616
Hematocrit, %			
Median (IqR)	26.9 (24.5–28.0)	25.0 (24.0–26.5)	0.2589
PT, s			
Median (IqR)	14.1 (12.4–16.1)	13.5 (12.5–15.5)	0.7724
PTT, s			
Median (IqR)	15.7 (12.6–16.6)	13.9 (12.4–14.9)	0.5089
Balanced saline solution, mL			
Median (IqR)	248.5 (226.5–309.5)	425.0 (362.5–553.4)	0.0009

*Calculated according to the formula: DBP + 1/3 (SBP-DBP).

*IqR* interquartile range, *bpm* beats per minute, *MBP* mean blood pressure, *PT* prothrombin time, *PTT* partial thromboplastin time.

### Primary endpoint

**Figure 1:**
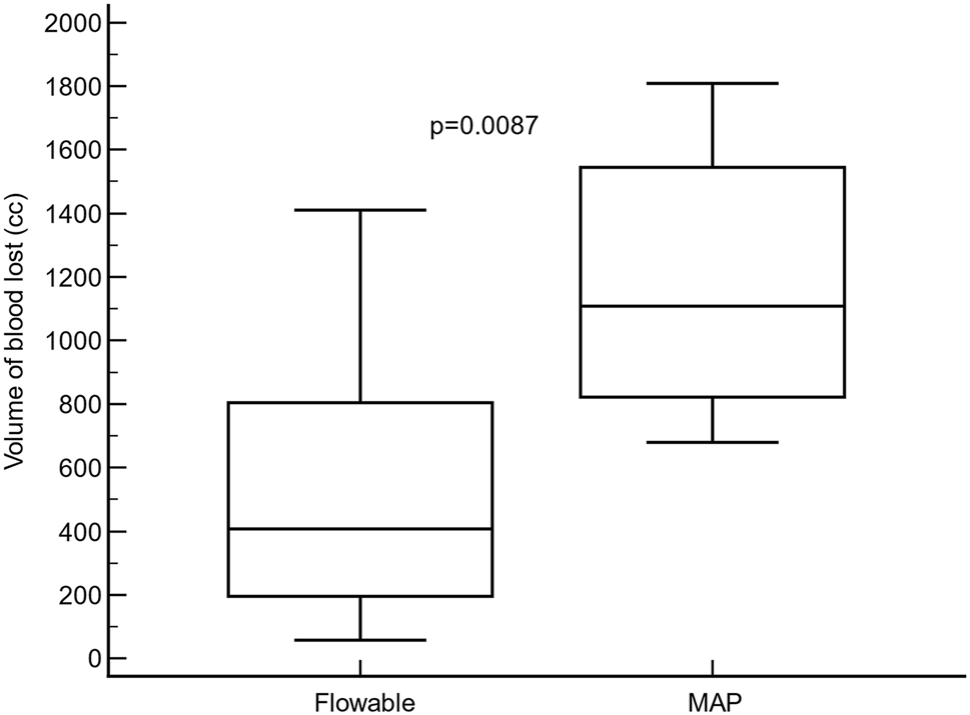
A comparison of the blood volume lost (cc) from injury to minute 120 between the flowable and the modified absorbable polymer (MAP) groups. Statistical significance was calculated by a Mann–Whitney *U* test.

The total volume of blood loss, from hepatic injury to minute 120, was significantly lower in the flowable group (407.5 cm^3^; IqR: 195.0–805.0 cm^3^) compared to the MAP group (1107.5 cm^3^; IqR: 822.5–1544.5 cm^3^) (Hodges–Lehmann median difference: − 645.0 cm^3^; 95% CI: − 1144.0 to − 280.0 cm^3^; p < 0.0087) (Fig. 1).

### Secondary outcomes

In the flowable group, the median (IqR) blood volume lost was 240.0 cm^3^ (120.0–415.0 cm^3^), from hepatic injury to minute 3; 70.0 cm^3^ (27.5–117.5 cm^3^) from minute 3 to minute 6; 27.5 cm^3^ (10.0–75.0 cm^3^) from minute 6 to minute 9; 20.0 cm^3^ (5.0–60.0 cm^3^) from minute 9 to minute 12; 22.5 cm^3^ (5.0–100.0 cm^3^) from minute 12 to minute 60; and 10.0 cm^3^ (1.3–10.0 cm^3^) from minute 60 to minute 120 (p < 0.0001, Friedman rank sum test). In the MAP group, the median (IqR) blood volume lost was 570.0 cm^3^ (480.0–660.0 cm^3^) from injury to minute 3; 115.0 cm^3^ (77.5–207.5 cm^3^) from minute 3 to minute 6; 107.5 cm^3^ (77.5–175.0 cm^3^) from minute 6 to minute 9; 85.0 cm^3^ (52.5–232.5 cm^3^) from minute 9 to minute 12; 50.0 cm^3^ (35.0–215.0 cm^3^) from minute 12 to minute 60; and 410.0 cm^3^ (35.0–53.8 cm^3^) from minute 60 to minute 120 (p < 0.0001, Friedman rank sum test).

**Figure 2:**
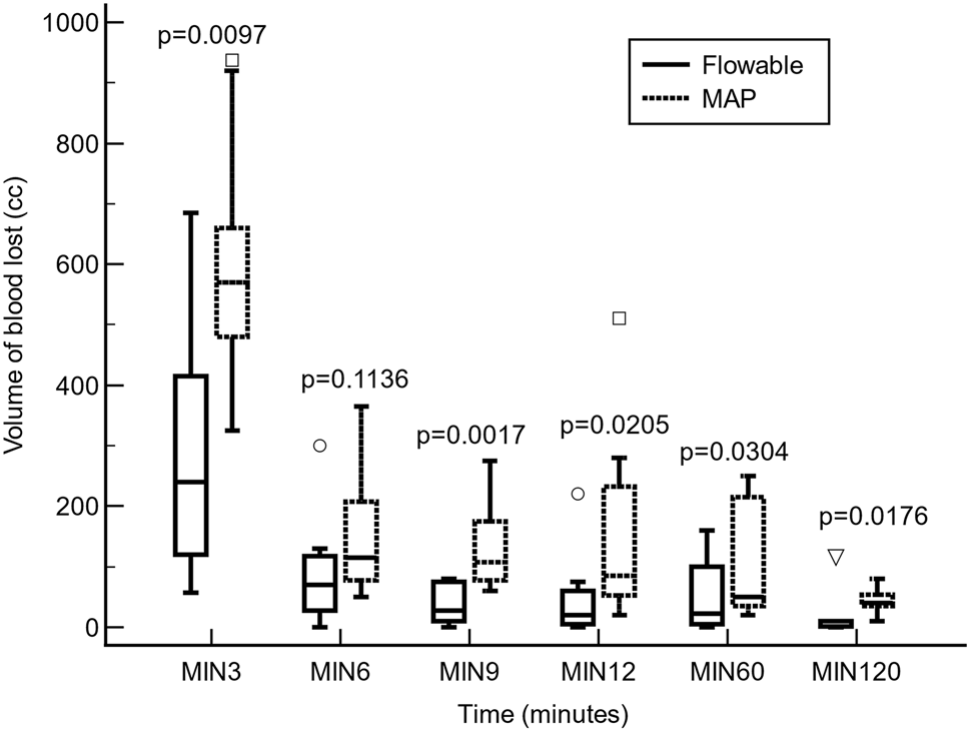
A comparison of the blood volume lost (cc) between the flowable and the modified absorbable polymer (MAP) groups. Statistical significance was calculated by a Mann–Whitney *U* test.

With the exception of the minute 6 measurement (Hodges–Lehmann median difference: − 57.0 cm^3^; 95% CI: − 155.0 to 10.0 cm^3^, p < 0.1136), the volume of blood lost was significantly lower in the flowable group compared with the MAP group from injury to minute 3 (Hodges–Lehmann median difference: − 327.5 cm^3^; 95% CI: − 490.0 to − 90.0 cm^3^, p < 0.0097); from minute 6 to minute 9 (Hodges–Lehmann median difference: − 80.0 cm^3^; 95% CI: − 140.0 to − 40.0 cm^3^, p < 0.0017); from minute 9 to minute 12 (Hodges–Lehmann median difference: − 60.0 cm^3^; 95% CI: − 200.0 to − 15.0 cm^3^, p < 0.0205); from minute 12 to minute 60 (Hodges–Lehmann median difference: − 35.0 cm^3^; 95% CI: − 175.0 to − 5.0 cm^3^, p < 0.0304); and from minute 60 to minute 120 (Hodges–Lehmann median difference: − 35.0 cm^3^; 95% CI: − 50.0 to − 10.0 cm^3^, p < 0.0176) (Fig. 2).

**Figure 3:**
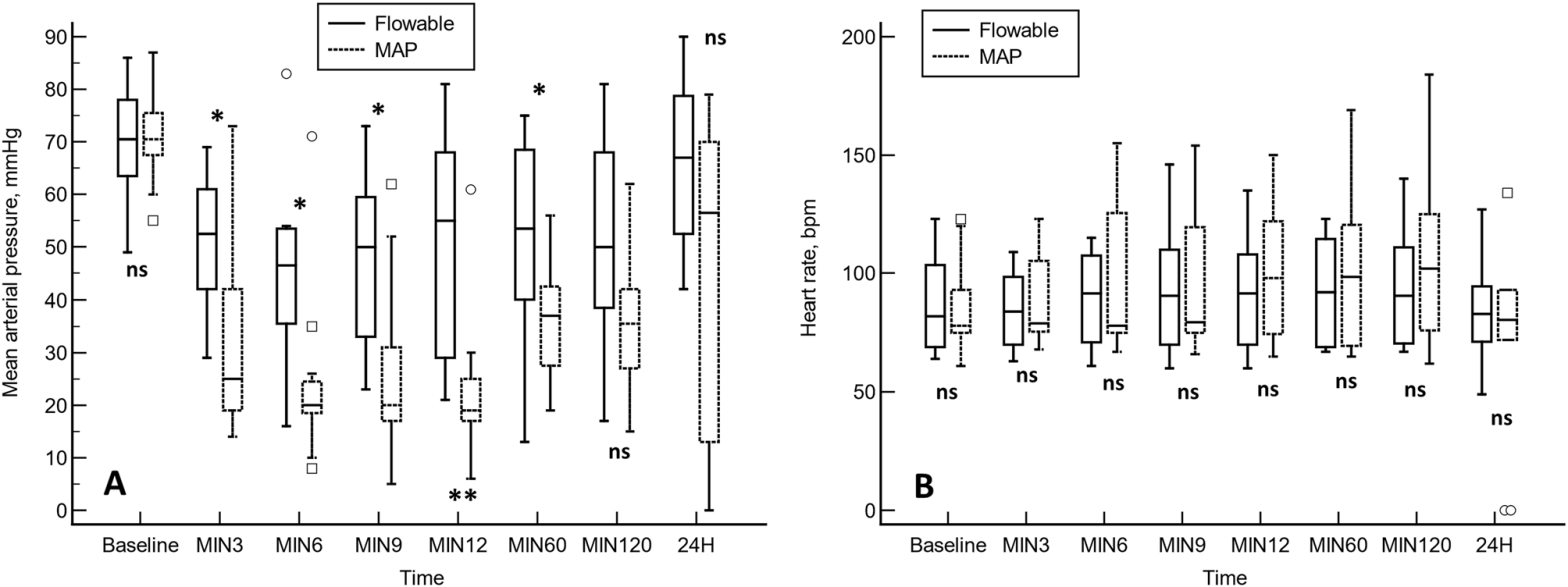
A comparison of the mean arterial pressure (**A**) and heart rate (**B**) between the flowable and the modified absorbable polymer (MAP) groups. Statistical significance was calculated by a Mann–Whitney *U* test. *p < 0.05. **p < 0.01. *Ns* not significant.

In the flowable group, the mean arterial pressure was significantly higher at minutes 3, 6, 9, 12, and 60 compared to the MAP group (Fig. 3A). The mean arterial pressure gradually recovered in the flowable group by 24 h, whereas in the MAP group, the mean arterial pressure was consistently below baseline values (Fig. 3A).

The heart rate remained stable throughout the study in both the flowable (Friedman test, p < 0.9983) and MAP groups (Friedman test, p < 0.7979), with no significant differences between the study groups at any of the time points measured (Fig. 3B).

**Figure 4:**
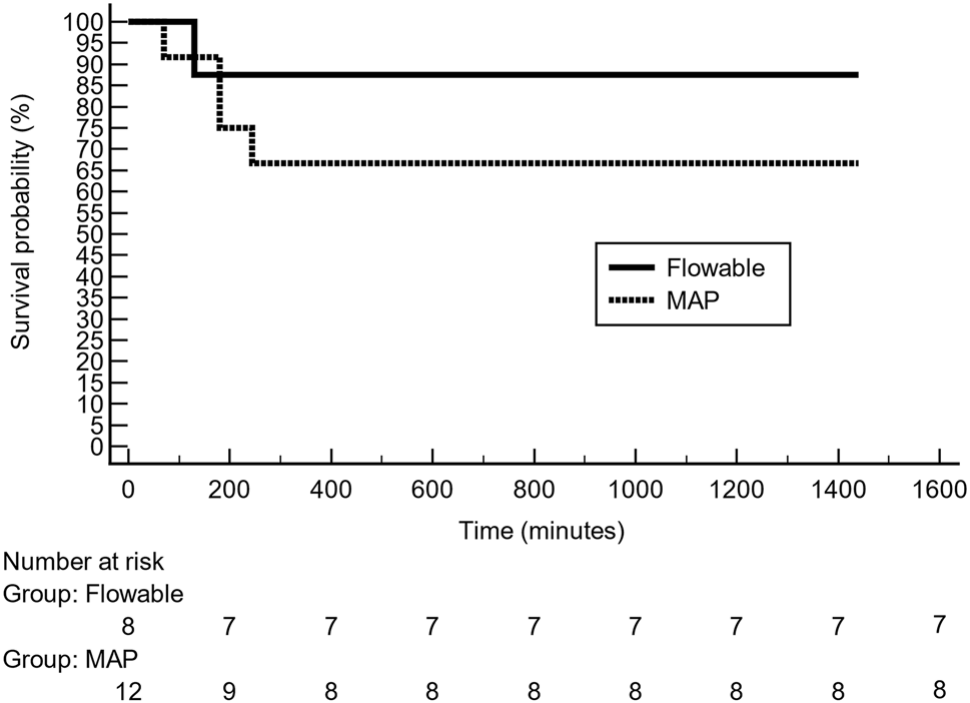
Kaplan–Meier survival curves for failure in the flowable group (solid line) and the modified absorbable polymer (MAP) group (dotted line). Death occurred in 1 (12.5%) flowable-treated pig and in 4 (33.3%) MAP-treated pigs (hazard ratio (HR) 0.42, 95% confidence interval 0.07–2.52); Logrank test p < 0.3395).

Kaplan–Meier survival analysis indicated similar rates of death between the study groups (hazard ratio: 0.41; 95% CI: 0.07–2.52); Logrank test p < 0.3395) (Fig. 4).

The application time was similar in the flowable (median: 28.0 s; IqR: 21.0–33.0 s) and in the MAP (median: 32.0 s; IqR: 29.0–37.5 s) groups (Hodges–Lehmann median difference: − 4.5 s; 95% confidence interval: − 12.0 to 3.0 s; p < 0.1747).

**Figure 5:**
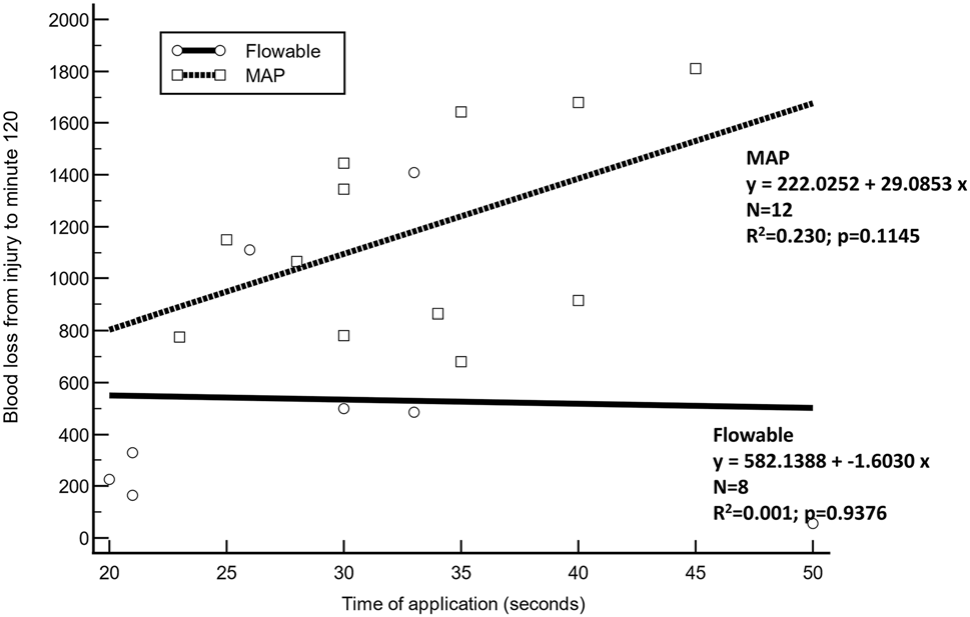
A comparison of the linear regression between the time needed for application and the volume of blood lost from injury to minute 120 between the flowable group (solid line) and the modified absorbable polymer (MAP) group (dotted line). Slope difference: − 30.69 + − 25.60; p < 0.2481.

Linear regression analysis did not show a significant correlation between the time needed for application and the volume of blood lost from injury to minute 120 in either the flowable group (R^2^ = 0.001, p = 0.9376, regression line slope − 1.6 cm^3^/s; where 95% CI ranged from − 49.7 to 46.5 cm^3^/s) nor in the MAP group (R^2^ = 0.230, p = 0.1145, regression line slope 29.1 cm^3^/s; where 95% CI ranged from − 8.4 to 66.6 cm^3^/s). Overall, there were no significant differences in the slopes between the two groups (mean difference, − 30.7; standard error, 25.6; p < 0.2481) (Fig. 5).

Similarly, there was not a correlation between the time needed for application and the volume of blood lost from injury to minute 12 in either the flowable group (R^2^ = 0.00, p < 0.9205, regression line slope − 0.0 cm^3^/s; where 95% CI ranged from − 0.03 to 0.03 cm^3^/s) nor in the MAP group (R^2^ = 0.26, p < 0.0900, regression line slope 0.01 cm^3^/s; where 95% CI ranged from − 0.00 to 0.02 cm^3^/s). Overall, there were no significant differences in the slopes between the two groups (mean difference: − 0.01, standard error, 0.01; p < 0.3322).

## Discussion

### Active or passive hemostatic agent?

Hemostasis may be defined as a multilayered cellular and molecular response that stops hemorrhage at the site of tissue injury. The capacity to improve hemostasis during surgical procedures is a key factor in preventing blood loss, reducing perioperative morbidity and surgery times, and improving surgical outcomes^17^. A variety of these hemostatic agents are available, and have been categorizing based on their mechanism of action. In general, hemostatic agents can be divided into active and passive hemostatic agents, depending on how they interact within a patient’s coagulation cascade, and both groups have utility in different procedures as adjunctive therapies for controlling surgical bleeding when conventional methods are inefficient or impractical^5^. Active hemostatic agents provide their mechanism of action at the end of the clotting cascade in a biological manner bypassing the initial steps and facilitating formation of the fibrin clot even when other aspects of the coagulation cascade may be dysfunctional. Whereas, passive hemostatic agents are most effective for minimal bleeding scenarios and rely on a patient having an intact coagulation cascade so they can activate platelets by providing a structure where platelets can aggregate and activate while relying on the patient’s intact coagulation cascade and ability to produce clotting factors to support clot formation^5^.

Various novel hemostatic agents have been developed for use in open and laparoscopic procedures^9,18,19^. In the active hemostatic group, we chose a flowable gelatin–thrombin-based product because it is a highly efficacious topical hemostatic agent^18,20^. Floseal is a well-known flowable agent composed of cross-linked hydrolyzed bovine gelatin (500–600 µm particles) and human thrombin (500 IU/mL)^14^.

In the current study, the evaluated passive hemostatic agent was PerClot. We chose this product because it has improved chemical characteristics over previous MAP and it has also shown to improve survival and blood loss when compared to a surgical technique (perihepatic packing) in swine^13^. PerClot is composed of modified absorbable polymer (MAP) granules that have a molecular structure that rapidly absorbs water, forming a gelled adhesive matrix^15^. It is a passive hemostatic agent that provides a mechanical barrier against further bleeding and results in the accumulation of platelets, red blood cells, and coagulation proteins (thrombin, fibrinogen, etc.) while providing a physical structure around which platelets can aggregate and promote the normal physiological clotting cascade^5,21^, and it has been designed as an adjunct hemostatic to control bleeding during surgical procedures or following traumatic injuries^17^.

### Which hemostatic agent works better alone?

To the best of our knowledge, this is the first study to compare PerClot, a passive hemostatic agent, with Floseal, an active hemostatic agent, in a novel and severe experimental liver injury model, without any other surgical maneuver. Other authors used hemostasis under greatly reduced flow conditions as the products were applied during a perihepatic packing or Pringle maneuver^22^. In one study, a hemostatic agent was used in addition to a surgical technique, making it very difficult to decipher whether the reduction of blood loss was due to the hemostatic agent, or the surgical technique, or a combination of both^23^.

Furthermore, if hemostasis is used as a single treatment, complications associated with other surgical techniques, such as increases in abdominal pressure^23,24^ or intra-abdominal abscess^25^ may be avoided.

#### Similarities

Both products performed sufficiently in this study of severe hemorrhage, as we did not find significant differences between the groups in terms of survival rates, heart rate, arterial pressure, or application time of the hemostatic agent (Figs. 3, 4). In addition, both hemostatic agents effectively controlled bleeding by 12 min.

In this study, the application time was used as a surrogate to measure ease of usability of the products. We did not find any significant differences in the speed of application between groups. Moreover, we did not find a correlation between the time taken to apply the hemostatic agent and the volume of blood lost at minutes 12 and 120, in either group (Fig. 5).

#### Differences

The primary endpoint of this study was the overall difference in blood loss, as it was hypothesized that the volume of blood loss could be a good parameter for analyzing hemostatic strength. We observed that Floseal had a stronger hemostatic effect compared with PerClot, due to the fact that the flowable group resulted in less blood loss (407.5 cm^3^) compared with the powder group (1107.5 cm^3^) (p < 0.0087). (Fig. 1).

Moreover, Floseal was faster than PerClot in achieving hemostasis, and its effect was more stable in the first few minutes. A hemostatic effect was observed as early as minute 3, giving Floseal a significant advantage over PerClot (Fig. 2). Later, at minute 6, both products seemed to equalize in their effect, and afterwards, at minutes 9 and 12, a significant difference was seen between the two groups (although the volume decreased). The speed and stability of coagulation could be related to the thrombin component in Floseal. The greater the thrombin concentration of the hemostatic agent, the faster the hemostatic effect was achieved.

Regarding blood loss, this study observed that most of the animals achieved hemostasis by minute 12, with continued bleeding in a few animals. In fact, five animals died (4 in the MAP group and one in the flowable group). Despite this difference, it might be said that both hemostatic agents statistically controlled bleeding by 12 min.

To the best of our knowledge, this is the first study to compare the rate of blood loss and survival after treatment with PerClot and Floseal in a severe swine liver injury model. The results are comparable with another study that used Floseal versus another MAP (Arista)^26^. Specifically, our results are consistent with this heparinized porcine hepatic abrasion model of capsular tears, in that Floseal provided greater control of bleeding, and the hemostatic success of the flowable agent was 17.5%, 40%, and 57.5% greater than that of the MAP agent (Arista) at 2, 5, and 10 min after application, respectively.

Also, there is an important difference between both products related on the implementation application.

The applicator of The PerClot may become blocked if the applicator becomes occluded (blocked) by blood. Therefore, it can should be administered as close as possible to the source of bleeding by scattering the product at a certain distance from the bleeding point so that the product is properly distributed^13,15^.

In contrast, we introduced the cannula of Floseal into the bloody injury, and there was no risk of rendering the system useless occluding the applicator tip.

Although there was a probability of embolism if the product was introduced into a major vessel, we did not observe any thrombosis or distant embolism in this study.

### Could it be applied in clinical practice?

Floseal has been used in various clinical scenarios, such as cardiac, thoracic, and nephrectomy surgeries^8,14,27^. In a prospective, randomized clinical trial of 309 patients with cardiac, vascular, or spinal surgery, the authors reported the control of bleeding at 3 min (85% vs. 48%, p < 0.001) compared to a thrombin-soaked gel foam, with a mean bleed control of 2.8 min. Moreover, in the cardiac surgery cohort, the control of bleeding was 77% vs. 0% at minute 3 (p < 0.0037) and 92% vs. 40% (p < 0.0057) at minute 10 in “severe bleeding”^28^. On the other hand, PerClot had fewer reportings compared to Floseal, but also observed a decreased time of hemostasis^29–31^. Our preclinical observations agree with the results of this clinical trial; both are efficient surgical hemostatic products, but Floseal is quicker and more stable.

### Characteristics of liver injury

The novel wound model implemented in this study was unique from others used previously, in that it was incisive, severe, and reproducible^32–37^. Additionally, it produces a standard wound that can be accurately compared across studies^13^.

### Limitations

Both PerClot and Floseal, are a sampling of the large variety of active and passive hemostatic agents currently available. Therefore, to further compare the clinical outcomes of active and passive hemostatic agents, new prospectives and randomized trials are needed.

Of note, this study used a novel, severe experimental liver injury model that caused massive hemorrhage. Therefore, caution must be exercised when applying these findings in clinical practice.

## Conclusions

According to the results of this study, both Floseal and PerClot hemostatic agents effectively controlled surgical bleeding in this severe liver injury model, although the Floseal provided quicker and better bleeding control.

## Data Availability

Data and material are available on request for ethical or legal reasons by contacting: franciscosanchez@healthgood.es.

## References

[1] Corral M, Ferko N, Hollmann S, Broder MS, Chang E (2015). Health and economic outcomes associated with uncontrolled surgical bleeding: A retrospective analysis of the Premier Perspectives Database. ClinicoEconomics Outcomes Res. CEOR.

[2] Ghadimi K, Levy JH, Welsby IJ (2016). Perioperative management of the bleeding patient. Br. J. Anesth..

[3] Stokes ME, Ye X, Shah M, Mercaldi K, Reynolds MW, Rupnow MFT, Hammond J (2011). Impact of bleeding-related complications and/or blood product transfusions on hospital costs in inpatient surgical patients. BMC Health Serv. Res..

[4] Shander A (2007). Financial and clinical outcomes associated with surgical bleeding complications. Surgery.

[5] Iannitti DA, Kim C, Ito D, Epstein J (2021). Impact of an active hemostatic product treatment approach on bleeding-related complications and hospital costs among inpatient surgeries in the United States. J. Med. Econ..

[6] Wright JD, Ananth CV, Lewin SN, Burke WM, Siddiq Z, Neugut AI, Herzog TJ, Hershman DL (2014). Patterns of use of hemostatic agents in patients undergoing major surgery. J. Surg. Res..

[7] Chapman WC, Singla N, Genyk Y, McNeil JW, Renkens KLJ, Reynolds TC, Murphy A, Weaver FA (2007). A phase 3, randomized, double-blind comparative study of the efficacy and safety of topical recombinant human thrombin and bovine thrombin in surgical hemostasis. J. Am. Coll. Surg..

[8] Echave M, Oyagüez I, Casado MA (2014). Use of Floseal, a human gelatine-thrombin matrix sealant, in surgery: A systematic review. BMC Surg..

[9] Chiara O, Cimbanassi S, Bellanova G (2018). A systematic review on the use of topical hemostats in trauma and emergency surgery. BMC Surg..

[10] Ragusa R, Faggian G, Rungatscher A, Cugola D, Marcon A, Mazzucco A (2007). Use of gelatin powder added to rifamycin versus bone wax in sternal wound hemostasis after cardiac surgery. Interact. Cardiovasc. Thorac. Surg..

[11] Lewis KM, Spazierer D, Urban MD, Lin L, Redl H, Goppelt A (2013). Comparison of regenerated and non-regenerated oxidized cellulose hemostatic agents. Eur. Surg. ACA Acta Chir. Austriaca.

[12] Tscholl V, Spann F, Moses J, Nagel P, Bellmann B, Biewener S, Amtenbrink M, Stroux A, Rillig A, Landmesser U, Roser M (2017). Prospective randomized study evaluating the effects of PerClot (Polysaccharide Hemostatic System) application in patients with high bleeding risk undergoing cardiac rhythm device implantation. Int. J. Cardiol..

[13] José SDVF, Luis DN, Juan GM, Antonio DP, Lidia SR (2023). Utility of microporous polysaccharide hemospheres in severe hepatic trauma: Experimental study of hemostatic strength and ease of use. Injury.

[14] Floseal Hemostatic Matrix, 10 mL. Instructions for use. Baxter Healthcare Corporation. Updated July 4, 2014. https://www.fffenterprises.com/assets/downloads/pi-Surgical%20Sealant_Floseal_Baxter.pdf. Accessed Aug 14, 2023.

[15] PerClot Polysaccharide Hemostatic System. Instruction for use. Starch Medical Incorporated. https://www.cryolife.com/wp-content/uploads/2019/08/PerClot-IFU-Rev.K-201901-.pdf. Accessed Aug 14, 2023.

[16] Madrid VA (1999). Aprovechamiento de los subproductos carnicos.

[17] Wu B, Song K, Gong Q, Zhan H, Chen W, Wang Z (2019). Perioperative outcomes and hospital costs associated with flowable gelatin hemostatic matrix for lumbar surgeries in real world hospital setting. J. Med. Econ..

[18] Gabay M, Boucher BA (2013). An essential primer for understanding the role of topical hemostats, surgical sealants, and adhesives for maintaining hemostasis. Pharmacotherapy.

[19] Tompeck AJ, Gajdhar AUR, Dowling M, Johnson SB, Barie PS, Winchell RJ, King D, Scalea TM, Britt LD, Narayan M (2020). A comprehensive review of topical hemostatic agents: The good, the bad, and the novel. J. Trauma Acute Care Surg..

[20] Lewis KM, Atlee HD, Mannone AJ, Dwyer J, Lin L, Goppelt A, Redl H (2013). Comparison of two gelatin and thrombin combination hemostats in a porcine liver abrasion model. J. Investig. Surg..

[21] Sheppard OO, Foje NA (2022). Topical coagulant agents. Surg. Clin. North Am..

[22] Pusateri AE, Holcomb JB, Kheirabadi BS, Alam HB, Wade CE, Ryan KL (2006). Making sense of the preclinical literature on advanced hemostatic products. J. Trauma Inj. Infect. Crit. Care.

[23] Meldrum DR, Moore FA, Moore EE, Haenel JB, Cosgriff N, Burch JM, Jack A (1995). Barney Resident Research Award. Cardiopulmonary hazards of perihepatic packing for major liver injuries. Am. J. Surg..

[24] Meldrum DR, Moore FA, Moore EE, Franciose RJ, Sauaia A, Burch JM (1997). Prospective characterization and selective management of the abdominal compartment syndrome. Am. J. Surg..

[25] Nicol AJ, Hommes M, Primrose R, Navsaria PH, Krige JEJ (2007). Packing for control of hemorrhage in major liver trauma. World J. Surg..

[26] Lewis KM, Atlee H, Mannone A, Lin L, Goppelt A (2015). Efficacy of hemostatic matrix and microporous polysaccharide hemospheres. J. Surg. Res..

[27] Ghimire S, Sarkar P, Rigby K, Maan A, Mukherjee S, Crawford KE, Mukhopadhyay K (2021). Polymeric materials for hemostatic wound healing. Pharmaceutics.

[28] Oz MC, Rondinone JF, Shargill NS (2003). Floseal matrix: New generation topical hemostatic sealant. J. Cardiac Surg..

[29] Beyer B, Leyh-Bannurah S-R, Isbarn H, Tennstedt P, Salomon G, Michl U, Heinzer H, Huland H, Graefen M, Budäus L (2013). 1023 Impact of a polysaccharide hemostat on bleeding complications and pelvic lymphocele rates after radical prostatectomy: Initial results of a prospective randomized trial. Eur. Urol. Suppl..

[30] Bruckner BA, Blau LN, Rodriguez L, Suarez EE, Ngo UQ, Reardon MJ, Loebe M (2014). Microporous polysaccharide hemosphere absorbable hemostat use in cardiothoracic surgical procedures. J. Cardiothorac. Surg..

[31] Jacobs, D. G., & Chrisas, A. B. (2020). Surgical techniques for managing hepatic injury—UpToDate. Updated February 24, 2022. https://www.uptodate.com/contents/surgical-techniques-for-managing-hepatic-injury. Accessed Aug 14, 2023.

[32] Jewelewicz DD, Cohn SM, Crookes BA, Proctor KG (2003). Modified rapid deployment hemostat bandage reduces blood loss and mortality in coagulopathic pigs with severe liver injury. J. Trauma.

[33] Cohn SM, Cross JH, Ivy ME, Feinstein AJ, Samotowka MA (1998). Fibrin glue terminates massive bleeding after complex hepatic injury. J. Trauma.

[34] Holcomb JB, Pusateri AE, Harris RA, Charles NC, Gomez RR, Cole JP, Beall LD, Bayer V, MacPhee MJ, Hess JR (1999). Effect of dry fibrin sealant dressings versus gauze packing on blood loss in grade V liver injuries in resuscitated swine. J. Trauma.

[35] Duggan MJ, Mejaddam AY, Beagle J, Demoya MA, Velmahosa GC, Alam HB, Rago A, Zugates G, Busold R, Freyman T, Sharma U, King DR (2013). Development of a lethal, closed-abdomen grade v hepato-portal injury model in non-coagulopathic swine. J. Surg. Res..

[36] Fonouni H, Kashfi A, Majlesara A, Stahlheber O, Konstantinidis L, Gharabaghi N, Kraus TW, Mehrabi A, Oweira H (2018). Hemostatic efficiency of modern topical sealants: Comparative evaluation after liver resection and splenic laceration in a swine model. J. Biomed. Mater. Res. Part B Appl. Biomater..

[37] Slezak P, Keibl C, Redl H, Labahn D, Gulle H (2020). An efficacy comparison of two hemostatic agents in a porcine liver bleeding model: Gelatin/thrombin flowable matrix versus collagen/thrombin powder. J. Investig. Surg..

